# Technology-Based Interventions to Promote the HIV Preexposure Prophylaxis (PrEP) Care Continuum: Protocol for a Systematic Review

**DOI:** 10.2196/33045

**Published:** 2022-03-08

**Authors:** Chenglin Hong, Laura S Abrams, Ian W Holloway

**Affiliations:** 1 Department of Social Welfare Luskin School of Public Affairs University of California, Los Angeles Los Angeles, CA United States; 2 Gay Sexuality and Social Policy Initiative Luskin School of Public Affairs University of California, Los Angeles Los Angeles, CA United States

**Keywords:** PrEP, pre-exposure prophylaxis, HIV, technology-based intervention, eHealth, mHealth, systematic review, HIV, HIV prevention

## Abstract

**Background:**

Preexposure prophylaxis (PrEP) is a promising biomedical intervention for HIV prevention. Researchers have proposed the PrEP care continuum to guide and evaluate PrEP implementation programs. Technology-based interventions (TBIs) have been widely used in HIV prevention and treatment programs, including for the promotion of the PrEP care continuum. The rapid development of new interventions using technology and electronic health methods emphasizes the need for a review of the effectiveness of these TBIs.

**Objective:**

The aim of this systematic review is to summarize the effectiveness and acceptability of TBIs used to promote the HIV PrEP care continuum.

**Methods:**

We will conduct a systematic literature search in PubMed, Embase, MEDLINE, PsycINFO, Web of Science, CINAHL, and the Cochrane Central Register of Controlled Trials following PRISMA (Preferred Reporting Items for Systematic Reviews and Meta-Analyses) guidelines. Only intervention studies (ie, studies meeting the criteria of randomized controlled trials or quasi-experimental studies) evaluating the effectiveness of TBIs will be included. We will search the National Institutes of Health Research Portfolio Online Reporting Tools (NIH RePORT) for interventions involving PrEP. At least 2 reviewers will independently screen and select the studies, extract the data, and evaluate the quality of the studies, and discrepancies will be resolved by a senior author. We will provide a narrative synthesis of the included studies and present details about the study populations, interventions, and PrEP-related outcomes of significance.

**Results:**

The protocol was registered in the International Prospective Register of Systematic Reviews (PROSPERO; CRD42021249562). As of August 2021, we have completed the initial search and identified 1213 records. Study screening and data extracting are in progress. We expect the results to be ready by summer 2022.

**Conclusions:**

The findings of this review will summarize successful experiences and lessons learned from the existing literature and therefore inform the design and implementation of intervention studies for PrEP care promotion.

**Trial Registration:**

PROSPERO CRD42021249562; https://www.crd.york.ac.uk/prospero/display_record.php?RecordID=249562

**International Registered Report Identifier (IRRID):**

DERR1-10.2196/33045

## Introduction

The efficacy and safety of preexposure prophylaxis (PrEP) for HIV prevention has been demonstrated through clinical trials among a range of at-risk populations, including men who have sex with men (MSM), people who inject drugs, and other high-risk populations [1-3]. The biomedical approach of daily oral antiretroviral medication is highly effective for preventing HIV when taken as prescribed [4,5]. To better facilitate the scale-up of PrEP and measure implementation progress, researchers proposed the PrEP care continuum (or PrEP care cascade, hereafter referred to as the PrEP continuum). Kelley et al [6] proposed a PrEP continuum framework to achieve protection for MSM that includes the following components: (1) at-risk MSM, (2) an awareness of and willingness to take PrEP, (3) access to health care, (4) a prescription for PrEP, and (5) adherence to effective PrEP. Nunn et al [7] expanded this framework as follows and included the additional step of retaining patients in PrEP care: (1) identifying individuals at highest risk for contracting HIV, (2) increasing HIV risk awareness among those individuals, (3) enhancing PrEP awareness, (4) facilitating PrEP access, (5) linking to PrEP care, (6) prescribing PrEP, (7) initiating PrEP, (8) adhering to PrEP, and (9) retaining individuals in PrEP care. Each step of the continuum represents a critical point for interventions, which can substantially increase the overall PrEP effectiveness at a population level. These frameworks guide researchers and practitioners to measure PrEP outcomes, and there are increasing numbers of published studies using the PrEP continuum to design and evaluate interventions to promote PrEP implementation worldwide [8-10].

In the past decade, electronic health and mobile health technologies, collectively referred to as technology-based interventions (TBIs), have seen exponential growth in the field of health care and health promotion. Examples of TBIs include disseminating health information on popular social media platforms, monitoring sleep duration and quality on wearable devices, sending push notifications to patients’ devices to support medication adherence, and using web-based chatrooms and meeting platforms for remote counseling [11]. These TBIs have several key features and functions that provide them with advantages over traditional health promotion interventions and programs, including their ability to reach a broader range of communities and individuals across diverse contexts, provide consistent services without the limitation of physical distance, and potentially be cost-effective and widely scalable once developed and adapted. Previous evaluation studies and meta-analyses have demonstrated and verified the effectiveness of such TBIs for supporting health behavior changes and disease management in several cases, including cardiovascular disease [12], diabetes [13], medication adherence [14], mental health, physical health [15], and sexual health including HIV and sexually transmitted infection prevention and care [16].

Compared to traditional HIV prevention interventions, TBIs can facilitate large-scale information dissemination and can effectively deliver just-in-time services and feedback to at-risk populations including MSM and people who inject drugs [17-19]. A recently published systematic review of TBIs related to HIV prevention and care cataloged published studies and other funded projects between 2014 and 2018 and found that there was a growing trend of TBIs and funded studies targeting various points on the HIV prevention and care continua with a substantial emphasis on education and behavior changes; however, there were no TBIs aiming to promote PrEP adherence and persistence [20]. From a nonsystematic review of various technologies used in HIV testing research, Romero et al [21] also noted that TBIs have the ability to increase outreach and HIV self-testing. To date, there is only one published review of TBIs involving PrEP; this review summarizes several telehealth programs aiming to improve PrEP availability, uptake, and adherence [22]. However, this review had some limitations. First, it is not clear whether it followed a systematic review guideline, and the authors only focused on telehealth approaches. Therefore, the results may not apply to all TBIs involving PrEP. In addition, similar to Maloney’s review [20], their analyses did not summarize the efficacy of each intervention and specifically evaluate which features of the interventions influenced each step of the PrEP continuum [22]. Finally, there was a lack of summary and evaluation on whether the TBIs were designed with guidance from a theoretical framework. In addition, little is known about the acceptability of and user experience associated with these TBIs and whether the interventions were scaled up and used in the real world after the completion of the study.

These considerations suggest the need for a comprehensive and rigorous review of the state of the science regarding TBIs and PrEP implementation. Therefore, the objectives of this systematic review are to (1) describe the use of TBIs in promoting steps along the PrEP continuum by population studied, (2) summarize the theoretical frameworks that have been used to inform and design the interventions, (3) summarize the evidence on and effectiveness of promoting steps along the PrEP continuum using the interventions, and (4) describe the acceptability of the TBIs among users, user experiences, and the sustainability of the intervention after study completion.

## Methods

### Protocol Registration and Design

This review was designed and reported in accordance with PRISMA (Preferred Reporting Items for Systematic Reviews and Meta-Analyses) guidelines [23,24]. A protocol outlining the review methods was registered in the International Prospective Register of Systematic Reviews (PROSPERO; CRD42021249562).

### Search Strategy

A systematic literature search was conducted using the following databases: PubMed, Embase, MEDLINE, PsycINFO, Web of Science, CINAHL, and the Cochrane Central Register of Controlled Trials. The search strategies were developed in consultation with a health science librarian and in accordance with previous reviews on similar topics [20,25]. The search terms were created using a combination of Medical Subject Headings, keywords, and phrases, including “PrEP” or “pre-exposure prophylaxis” in combination with, but not limited to, any of the following: “eHealth”, “mHealth”, “smartphone”, “mobile phone”, “mobile application”, “app”, “internet”, “text messages”, “social media”, “dating app”, “wearable device”, “website”, and “technology”. A manual search will be conducted using the reference lists of included publications and using Google Scholar to find other relevant studies. In addition, we will search the National Institutes of Health Research Portfolio Online Reporting Tools (NIH RePORT) for interventions still underway and contact the principal investigators for a basic summary of the interventions. Multimedia Appendix 1 provides details of the search strategy and terms used.

#### Eligibility Criteria

We will include published records of intervention studies (eg, randomized trials and quasi-experimental studies) that compare outcomes for individuals who participated in the TBI to any control group. As PrEP was first approved by the US Food and Drug Administration for HIV prevention in 2012, we used this year for the start of the search. We will exclude articles published in languages other than English and those that did not have a PrEP-related outcome as one of the primary outcomes. Study protocols and reports of ongoing trials will also be excluded. We will only include studies with original data; therefore, nonempirical studies such as letters, perspectives, and editorials will not be included.

#### Study Population

There are no restrictions on the sociodemographic characteristics of the study participants in the intervention studies.

#### Interventions

To date, there is no consensus on the definition of TBIs and what interventions fall under this category. We first created a list of TBIs and their features based on the extant literature and iteratively refined this list based on a review of the included studies. Given the broad definition, we included studies in which an intervention used information technology for service delivery to facilitate one or multiple steps of the PrEP continuum. These interventions include, but were not limited to, mobile apps, social media, web-based products, SMS text messaging, web-based chatrooms and quizzes, push notifications, and interactive geolocation maps.

#### Outcomes

The included studies must report at least one PrEP-related outcome. Although the previous PrEP continuum consists of multiple steps, this review will primarily focus on PrEP awareness, willingness to take PrEP, PrEP uptake, and PrEP adherence [8,10]. Although these broad definitions were summarized from the existing literature, we will extract how each study defined and measured these outcomes. Generally, PrEP awareness will be defined as having heard of PrEP and having accurate knowledge of PrEP efficacy for HIV prevention. Willingness to use PrEP is the hypothetical willingness and perceived acceptability of using PrEP in the future. PrEP uptake is the initiation and actual use of PrEP among the study participants. Although alternative PrEP options, such as long-acting injectable PrEP, are under development, this review will only focus on oral PrEP (Truvada, Descovy, and generics [26]). PrEP adherence is the level of medication adherence sufficient for the prevention of HIV acquisition. We will consider both daily and “on-demand” dosing (taking PrEP only when individuals are at risk for getting HIV is known as on-demand PrEP, or the “2-1-1 schedule,” which means taking 2 pills between 2 and 24 hours before sexual intercourse, 1 pill 24 hours after the first dose, and 1 pill 24 hours after the second dose [27]) if the articles clearly demonstrated how adherence was defined and measured in their studies. This could include self-reported days of missing the pills in the months prior to the study or lab results of tenofovir-diphosphate concentrations in dried blood spot samples [28]. All PrEP-related outcomes must be assessed and reported quantitatively (eg, using rates, percentages, or odds ratios).

Secondary outcomes include users’ experiences, acceptability of the TBI, and satisfaction rates. User acceptability and satisfaction can be assessed using multiple methods, including exit interviews or standardized questionnaires or scales, including the Computer Usability Satisfaction Questionnaire [29] and the Health Information Technology Usability Evaluation Scale [30].

#### Study Selection and Screening

We will import all the search records to Covidence (Veritas Health Innovation Ltd), a software that automatically removes duplicate studies. The remaining studies will be screened under blinded conditions by at least 2 independent reviewers. Article titles and abstracts will first be screened for eligibility, and any studies that meet the eligibility criteria or those that the reviewers cannot decide to include or exclude will progress to the full-text review. Next, 2 independent reviewers (CH and another reviewer) will screen the full texts of the articles to determine whether they will be included in the review and data extraction. The results of each step will be compared, and any inconsistency or conflict will be resolved through discussion or arbitration from a senior author (IWH) until a consensus is reached.

#### Data Extraction and Analysis

A standardized data extraction form will be created to enter the data from the included articles. A total of 2 reviewers will extract the data independently (CH and another reviewer), and the results will be compared and discussed until a consensus is reached. For each selected article, we will record the general study characteristics and intervention-specific data. Study characteristics include study authors, year of publication, study location, participants’ sociodemographic characteristics, study design, recruitment methods, and sample size. Intervention-specific data include the name of the study and a brief description of the intervention, study design, type of technology used for the intervention (eg, internet or social media), theoretical framework used to guide the intervention design (if applicable), comparator used, PrEP continuum domain that the study aimed to improve, and quantitative data on the comparisons between the intervention and the control group (eg, changes in percentages, rates, and odds ratios). Information on whether the intervention continued to be implemented after the study completion will be extracted from the included studies. The reviewer (CH) will contact the corresponding authors for clarification and further information where study details are missing or unclear. The studies will be categorized and grouped according to the type of intervention and the PrEP continuum domain that was targeted.

#### Risk of Bias Assessment

The quality of the studies will be assessed using the Cochrane Risk of Bias Tool for randomized controlled trials [31]. The domains being assessed include random sequence generation, allocation concealment, blinding of participants, blinding of personnel, blinding of outcome assessment, incomplete outcome data, selective reporting, and other sources of bias. All included studies will be scored as having a low, high, or unclear risk of bias.

## Results

As of August 2021, we have completed the preliminary searches on the databases previously listed. A total of 1213 records were identified. Eligibility screening and data extraction are ongoing. It is anticipated that the final review will be completed and submitted for publication in summer 2022.

## Discussion

The emergence of TBIs in public health research has created opportunities for health promotion and disease prevention. A growing number of intervention studies have investigated the effect of TBIs in supporting the PrEP continuum among various at-risk populations. This systematic review will provide an overview of these TBIs and describe the evidence supporting their use and their efficacy. The review will highlight which kind of interventions have been implemented and which PrEP continuum step(s) are primarily targeted. Furthermore, this review will be the first to summarize the theoretical frameworks used for informing TBI design. The findings of this review will summarize successful experiences and lessons learned from published literature, conference abstracts, NIH RePORT, and unpublished data. The findings will also inform the design and implementation of future studies on PrEP care promotion.

## Appendix

Multimedia Appendix 1 The search strategy used to find articles in PubMed.

## Abbreviations

MSM: men who have sex with men
NIH RePORT: National Institutes of Health Research Portfolio Online Reporting Tools
PrEP: preexposure prophylaxis
PRISMA: Preferred Reporting Items for Systematic Reviews and Meta-Analyses
PROSPERO: International Prospective Register of Systematic Reviews
TBI: technology-based interventions
UCLA: University of California, Los Angeles
