# Supplementary material for: Technology-Based Interventions to Promote the HIV Preexposure Prophylaxis (PrEP) Care Continuum: Protocol for a Systematic Review
Source: JMIR Res Protoc. 2022 Mar 8;11(3):e33045. doi: 10.2196/33045 (PMC8941443; doi:10.2196/33045)
Supplement: Multimedia Appendix 1 [file resprot_v11i3e33045_app1.docx]

**Multimedia Appendix 1.** The search strategy used to find articles in PubMed.

PubMed search keywords

("Computer systems"[mesh] OR "Educational Technology"[mesh] OR "Text Messaging"[mesh] OR "Cell Phone"[mesh] OR "software"[mesh:noexp] OR "telemedicine"[mesh] OR "online systems"[mesh:noexp] OR "reminder systems"[mesh] OR "information systems"[mesh:noexp] OR "user-computer interface"[mesh] OR "mobile applications"[mesh] OR "video games"[mesh] OR "videotape recording"[mesh] OR "videodisc recording"[mesh] OR "computer-assisted instruction"[mesh] OR technology[tiab] OR technologies[tiab] OR computer*[tiab] OR smartphone*[tiab] OR ehealth[tiab] OR mhealth[tiab] OR telehealth[tiab] OR texting[tiab] OR text messag*[tiab] OR sms[tiab] OR cell phone*[tiab] OR mobile phone*[tiab] OR cellular phone*[tiab] OR mobile device*[tiab] OR ipad*[tiab] OR tablet[tiab] OR tablets[tiab] OR "mobile app"[tiab] OR "mobile apps"[tiab] OR mobile application*[tiab] OR "web based"[tiab] OR "network based"[tiab] OR audiovisual[tiab] OR multimedia[tiab] OR social media [tiab]OR video[tiab] OR videos[tiab] OR videorecord*[tiab]))

AND

(pre-exposure prophylaxis OR preexposure prophylaxis OR "PrEP" OR HIV pre-exposure prophylaxis OR HIV preexposure prophylaxis OR pre-exposure antiretroviral prophylaxis OR preexposure antiretroviral prophylaxis OR pre-exposure chemoprophylaxis OR preexposure chemoprophylaxis OR anti-HIV prophylaxis)
